# Dataset for DME oxidation behavior of commercial Pt/Pd-series diesel oxidation catalysts

**DOI:** 10.1016/j.dib.2025.112380

**Published:** 2025-12-11

**Authors:** Tom Padeken, Alexander Lampkowski, Peter Mauermann, Werner Willems, Christian Nederlof, Björn Franzke, Stefan Sterlepper, Bastian Lehrheuer, Stefan Pischinger

**Affiliations:** a Chair of Thermodynamics of Mobile Energy Conversion Systems, RWTH Aachen University, Forckenbeckstrasse 4, 52074 Aachen Germany; b TEC4FUELS GmbH, Kaiserstraße 100, 52134 Herzogenrath, Germany; c DAF Trucks N.V., Hugo van der Goeslaan 1, 5643 TW Eindhoven, The Netherlands; d FEV Europe GmbH, Neuenhofstr. 181, 52078 Aachen, Germany; e FSC – The Fuel Science Center, RWTH Aachen University, Schinkelstrasse 8, 52062 Aachen, Germany; f ACA – Center for Automotive Catalytic Systems Aachen, Germany

**Keywords:** Dimethyl ether, DME, Diesel oxidation catalyst, Exhaust gas aftertreatment, Secondary emission, Formaldehyde, Synthetic gas test bench

## Abstract

This article presents data collected during a measurement campaign on a synthetic gas test bench (SGB) from the Chair of Thermodynamics of Mobile Energy Conversion Systems (TME). The measurement campaign consists of 23 light-off experiments. Five state-of-the-art diesel oxidation catalysts (DOCs) with varying platinum and palladium formulations were used. The objective of this study is to gain insight into the oxidation behavior of dimethyl ether (DME) and its influence on other reactions during exhaust gas aftertreatment. A total of five distinct gas compositions were utilized in order to emulate both reduced and model conditions, as well as realistic conditions for the simulation of DME and DME/diesel fuel blend exhaust gas. DME simulates the slipped hydrocarbon emission for a pure DME combustion in an internal combustion engine and Propene is incorporated as a constituent in order to simulate the combustion of a DME/diesel fuel blend.

The dataset presented in this article encompasses comprehensive test bench data, including temperature readings, mass flow controller metrics, and gas analytical values. This data has been curated primarily for analyzing the light-off temperature ramp, with additional processing conducted to establish benchmark values for light-off behavior. The datasets also encompassing the time frame from pre-conditioning to post-conditioning. Post-processing of the SGB data allows for the calculation of gas concentrations at the catalyst inlet. Measured gas analytics have undergone zero-point and time corrections to account for drift and gas flow runtime variations. The analysis of exhaust gases includes measurements of fundamental components expected during complete oxidation. The diverse array of exhaust gas analyzers that also captures additional hydrocarbon species associated with secondary reaction pathways and intermediates. The data provided in this article offers valuable insights to researchers and industry professionals regarding the oxidation behavior of DME, its co-oxidation effects, and the formation of secondary emissions.

**Table:** Specifications Table.

Subject	Engineering & Materials science
Specific subject area	Catalyst characterization for exhaust gas aftertreatment of model dimethyl ether and dimethyl ether/diesel fuel blend exhaust gas
Type of data	Data tables (.csv format) Usage note (.txt format) Raw, Analyzed, Processed
Data collection	The data was collected through experiments at the self-designed synthetic gas test bench (SGB) and a self-made post processing script, based in visual basic. The test bench data comprises of temperature, calculated inlet concentrations and measured outlet concentrations. The measured gas analytics have undergone zero point and time correction to counter drifts and gas flow runtimes. The vast equipment for test bench operation and data acquisition, as well as gas analytic equipment can be found in the experimental design, materials and methods section.
Data source location	Chair of Thermodynamics of Mobile Energy Conversion Systems, RWTH Aachen University, Forckenbeckstr. 4, 52,074 Aachen, Germany.
Data accessibility	Repository name: Zenodo Data identification number: https://doi.org/10.5281/zenodo.17807380 Direct URL to data: https://zenodo.org/records/17807380

## 1 Value of the Data

• The data set gives valuable insigt in the DME conversion behavior in different settings, for different diesel oxidation catalysts, derived from series application. The impacts of co-oxidation with proplylene and the influences CO, NO and CO_2_ can be derived from this dataset. Also, the formation of secondary emissions during the oxidation and co-oxidation of DME, is reported within the data, supporting the research question of DME/Diesel-fuel blend combustion.
• The data is also useful to show the effect of DME on the NO oxidation, as well as to show a potential hysteresis of all reactions during the light-off and a consecutive temperatature decrease.
• The data may be used by researchers and industry professionals to clibrate and adapt simulations. Map-based simulation approaches of driving cycles could be adapted, regarding the new HC conversion behavior, as well as the secondary emissions formation to gain insight on potential performance within targeted emission regulations. This, among the interpretation of the concentration profiles gives valuable guidance in the design process of exhaust gas afterstreatment systems for DME containing exhaust gases, starting from the pre-development stage.

## 2 Background

These datasets were created as part of the DMEplusX project and the Fuel Science Center to investigate the impact of DME-containing exhaust gases on series production exhaust gas aftertreatment systems. The presented datasets show the initial screening of diesel oxidation catalysts (DOCs) using synthetic gas on a test bench with DME-containing exhaust gases. The project's global aim is to investigate the gradual introduction of renewable fuels into the legacy fleet by mixing DME with diesel fuel. This is achieved while maintaining most of the original 'diesel' setup and minimising retrofitting efforts. Thus, the exhaust aftertreatment is exposed to DME-containing exhaust gas, and its impact is evaluated. The sub-task presented in this article deals with the DOCs that were involved in the project. The first objective of the DOC test campaign on the synthetic gas test bench is to analyse conversion behaviour in different gas mixtures to simulate model and more realistic exhaust gas compositions. The second objective is to detect the secondary emissions formed as a result of DME oxidation and co-oxidation.

## 3 Data Description

The dataset includes results from 20 experiments across DOCs 1–5, for four different gas matrices:

1. A reduced gas matrix for pure DME oxidation with only O_2_.
2. A realistic gas matrix for DME oxidation under actual exhaust gas conditions, incorporating CO, NO, and CO_2_ into the reduced mix to simulate DME combustion exhaust.
3. A reduced gas mix for pure DME and propene co-oxidation with only O_2_.
4. A realistic gas matrix for DME and propene co-oxidation under genuine exhaust conditions, again integrating CO, NO, and CO_2_ to simulate a DME/diesel exhaust scenario.

Additionally, three experiments involving DOCs 1, 3, and 5 are included with gas mixture 5:

5. This realistic gas mix excludes any hydrocarbons (HC) to assess their influence on NO oxidation.

The dataset under consideration encompasses a total of 23 experiments, each of which is delivered in .csv format (decimal separator: . [dot];symbol for digit groupings: [space]; list separator:, [comma]). The file naming convention employed is as follows: The abbreviation “DOC_X_GM_Y.csv” is employed herein:

• X indicates the respective DOC number (1–5),
• Y denotes the corresponding gas matrix (GM), ranging from 1 to 5.

The datasets for exhaust gas analytics encompass key components such as DME, O_2_, CO, CO_2_, NO, along with additional HC species, which may be generated during the oxidation processes of DME and propene. The notable components include formaldehyde, formic acid, methanol, and nitric oxide. N_2_ is utilized as a carrier gas. Alongside this information, different relevant values of controls, temperatures and post processing are included in the dataset, which are published by Padeken et al. [1]. The individual datasets of each experiment are summarized for each DOC and zipped. The readme.txt specifies the formatting of the dataset within that publication and the DOC_sample_loading_and_test.csv gives the information regarding the DOCs composition and the gas matrices used.

All parameters and values are listed and described in Table 1 where its floating variables X and Y for the different species are described below.

**Table 1:** Variable description within the dataset.

Variable	Floating Variable	Unit	Description
dt	-	s	Recording time frame
time	-	s	Time
Start_LOF	-	s	Timestamp of the start of the light-off temperature ramp
time_LOF	-	s	Time with the origin at the start of the light-off temperature ramp
Start_LOT	-	s	Timestamp of the start of the light-out temperature ramp
time_LOT	-	s	Time with the origin at the start of the light-out temperature ramp
Aft_Cat	-	-	Variable of the catalyst bypass (0 = bypass open; 1 bypass closed)
stage	-	-	Stage of operation of the planned experiment
st_dos	-	-	Variable if gas is dosed (1 = dosing on; 0 = dosing off)
vol_Cat	-	l	Volume of the catalyst sample
volFlow_tot	-	l/min	Total volume flow
r_SpaceVelo	-	1/h	Gas related hourly space velocity
p_befCat	-	bar	Pressure before the catalyst sample
T_befCat	-	°C	Temperature before the catalyst sample
T_CatX	X	°C	Temperature within the catalyst sample, position *X* = 1 - 5
T_Cat	-	°C	Average temperature over all individual temperatures within the catalyst sample
conc_fidHC	-	ppm	Measured total hydrocarbons w/ FID (C3 base)
conc_dosHC_C3	-	ppm	Dosed total hydrocarbons on C3 base
conc_dosX	X	ppm	Dosed concentrations of the respective species (*X* = specified below)
conc_X	X	ppm	Measured concentrations of the respective species (*X* = specified below)
molFlow	-	mol/s	Total molar flow rate
molFlow_dosX	X	mol/s	Dosed molar flow rates of the respective species (*X* = specified below)
massFlow_dosX	X	g/s	Dosed mass flow rates of the respective species (*X* = specified below)
molFlow_X	X	mol/s	Measured molar flow rates of the respective species (*X* = specified below)
massFlow_X	X	g/s	Measured mass flow rates of the respective species (*X* = specified below)
X_Y	Y	-	Conversion rate of the respective species *Y* = specified below
Y_NO2		-	NO_2_ yield
S_NO2		-	NO_2_ selectivity
Y_N2O		-	N_2_O yield
S_N2O		-	N_2_O selectivity
TA_X_B_LOF	A / B	°C	Temperature at the conversion rate *A* = 10, 50, 80 % of the respective species (*B* = C_2_H_6_O, C_3_H_6_, CO, CH_4_) during the light-off temperature ramp
T50_X_B_LOT	B	°C	Temperature at the conversion rate 50 % of the respective species (*B* = C_2_H_6_O, C_3_H_6_, CO, CH_4_) during the light-out temperature ramp

Floating variable X = CO, CO_2_, NO, NO_2_, NO_x_, N_2_O, NH_3_, O_2_, C_3_H_8_, H_2_O, CH_4_, C_3_H_6_, H_2_, C_2_H_2_, C_2_H_4_, C_2_H_6_, HNCO, C_2_H_4_O, C_3_H_8_O_2_, CH_2_O_2_, C_7_H_8_, C_4_H_6_, CH_2_O, C_2_H_6_O, CH_4_O

Floating variable Y = C_2_H_6_O, CO, C_3_H_6_, H_2_, NO. NO_2_, CH_4_, O_2_, C_2_H_2_, C_2_H_4_, C_7_H_8_, CH_4_O, C_2_H_4_O_2_, C_3_H_8_O_2_ CH_2_O, C_2_H_4_O

## 4 Experimental Design, Materials and Methods

### 4.1 Synthetic gas test bench setup

The development of the Synthetic Gas Test Bench (SGB) was undertaken in-house by the Chair of Thermodynamics for Mobile Energy Conversion Systems. The primary function of this test stand is to investigate monolithic catalyst samples [[2], [3], [4]], but also facilitates the examination of granule samples. This allows the investigation starting with washcoats throughout the entire catalyst development process [5], as well as conducting filtration studies [6], due to the precision gas dosing and analysis.

**Fig. 1:**
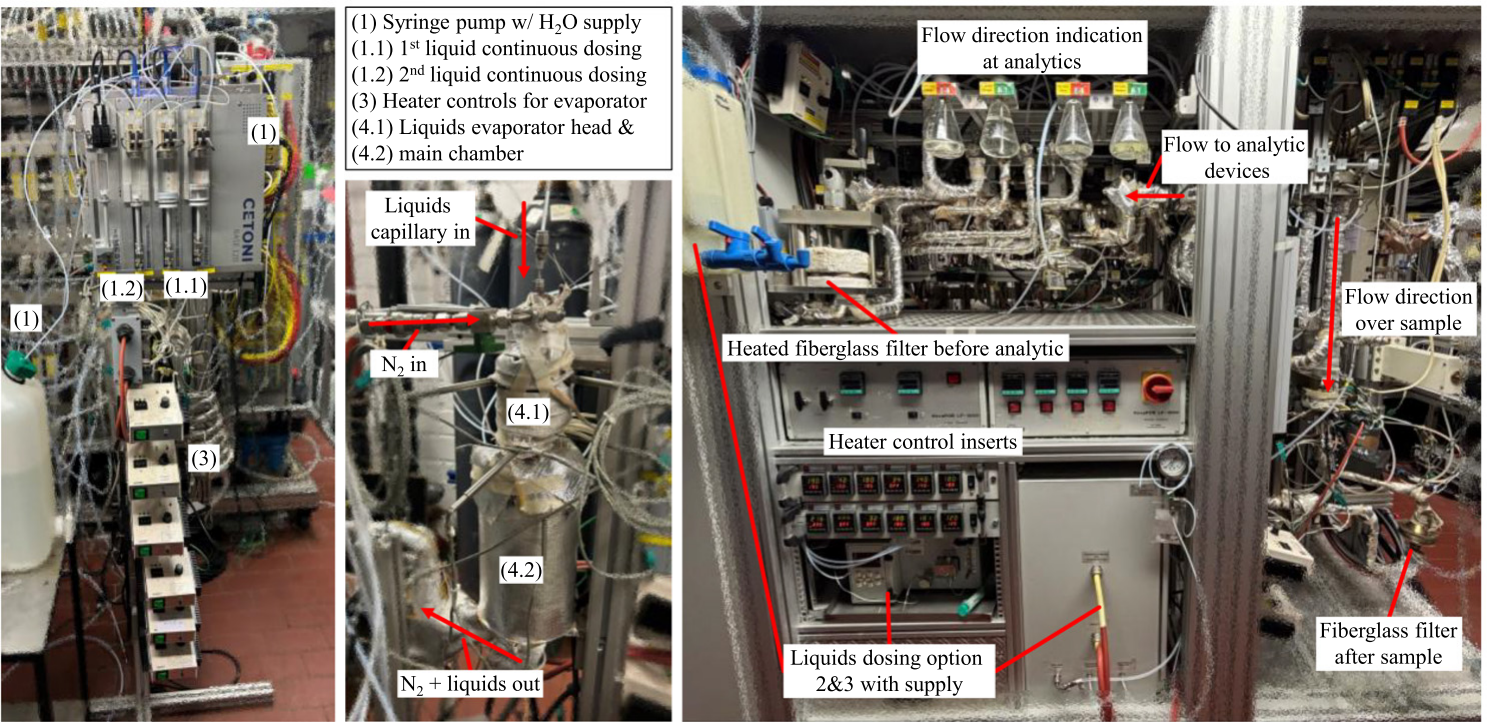
Setup of the synthetic gas test bench liquids dosing setup.

Gas dosing is managed through gas cylinders containing individual components. In order to achieve the desired concentrations during experiments, mass flow controllers (MFCs) from Brooks LLC are utilized. In order to guarantee that the minimum degree of leakage is achieved when a MFC is closed, these controllers are coupled with solenoid valves from Bürkert GmbH & Co. KG. The MFCs operate under a pre-pressure of p = 5 bar, and a maximum of 32 MFCs can be employed simultaneously. A wide selection of additional MFCs from the MFC database enables precise dosing of multiple components while selecting space velocity, thus allowing realistic and model-like exhaust matrices to be simulated. Liquid components, including water and liquid hydrocarbons, are vaporized in a custom-designed and built evaporator and introduced into the experiments alongside a nitrogen gas stream. The liquids are administration by a precision syringe pump from Cetoni GmbH. The Nemesys S modules employed in this system enable the continuous dosing of two components, while discontinuous operation allows the dosing of up to four liquid components. Fig. 1 illustrates this liquids dosing setup on the two images on the left. In addition to the syringe pump, a S 9425 HPLC Pump System from SRI Instruments GmbH can administer fluids up to a pressure of p = 400 bar as a second dosing option, as well as a HovaPOR LF-1200 evaporation system from IAS GmbH as the third option. Both depicted next to each other in Fig. 1 on the right, where also the flow direction over the sample is also marked, which has been published and described by Lampkowski et al. [3]. The pressure at the catalyst sample is monitored via a pressure sensor PAA-33X from KELLER pressure, with the range of p_abs_ = 0…5 bar.

**Fig. 2:**
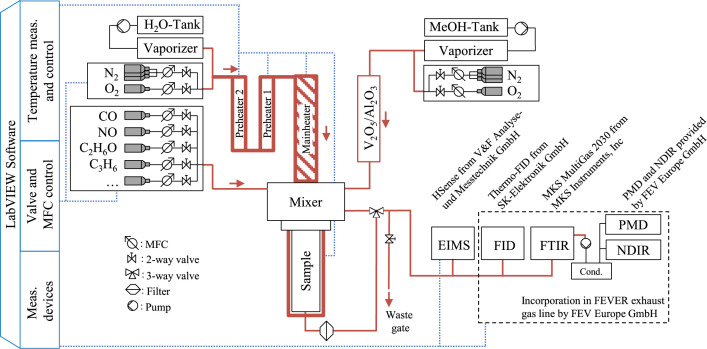
Schematic of the Laboratory gas test bench.

As illustrated schematically in Fig. 2, the test stand incorporates a series of heaters, including two preliminary heaters and a primary heater, which convectively heat the catalyst sample. The reactor is also heated to the temperature set point in order to minimize thermal losses within the sample.

Also as depicted in Fig. 1 and Fig. 2, Whatman fibreglass filter from Cytiva are positioned subsequent to the catalyst sample. This configuration is intended to prevent the potential migration of catalyst material through the test bench in the event of failure, thereby mitigating the risk of its accumulation within the test bench. The gas analytics are in addition, beyond that also equipped with a second filter. The measurement equipment, as illustrated in Fig. 2, will be discussed in the following section on the light-off procedure. In addition, the test bench is upgraded with additional equipment that enables the in-situ production of formaldehyde from liquid-dosed methanol. For this process, a V_2_O_5_/Al_2_O_3_ catalyst is installed in a bypass after the evaporator. This process allows the selective reaction of methanol to formaldehyde with the addition of O_2_ [7,8].

The test stand, in its entirety, is composed of titanium, inclusive of the sample holder (canning), which also allows the sequential insertion of multiple monolithic samples [9]. Further details regarding the preparation of monolithic samples can be found in the section on catalyst specifications and sample preparation.

### 4.2 Light-Off procedure

The objective of the present experiments is to ascertain how the various catalysts convert DME at elevated temperatures under the specified conditions. These experiments are designated as light-off experiments, as the light-off can be described as the conversion X_HC_ = 50 % of the dosed HC species. The DME conversion is calculated according to the following equation, with the DME Inlet concentration conc_dosC2H6O and the outlet concentration conc_C2H6O:

(1) $$X\_DME=\;\frac{conc\_dosC2H6O-\;conc\_C2H6O}{conc\_dosC2H6O}$$

Analougus to the conversion rate of DME, the conversion rates of the other species (CO, C_3_H_6_, H_2_, NO. NO_2_, CH_4_, O_2_, C_2_H_2_, C_2_H_4_, C_7_H_8_, CH_4_O, C_2_H_4_O_2_, C_3_H_8_O_2_ CH_2_O, C_2_H_4_O) are calculated in the same manor. For the gas matrices, containing NO and targeting the NO oxidation, the yield Y and selectivity S of NO_2_ and N_2_O are calculated on the example of N_2_O:

(2) $$Y\_N2O=\;\frac{2\;*\;\left(conc\_N2O\;-conc\_dosN2O\right)}{conc\_dosNOx\;}$$

(3) $$S_{N2O}=\;\frac{2\;*\;\left(conc_{N2O}-conc_{dosN2O}\right)}{conc_{dosNOx}-conc_{NOx}}$$

All light-off experiments displayed herein were executed at a gas-related space velocity of GHSV = 120,000 h⁻¹, with a constant water concentration of c_H2O_ = 8 vol.- % in N₂ as the carrier gas. The experiments are carried out with a DME concentration of c_DME_ = 500 ppm in the presence of c_O2_ = 10 vol.- % oxygen for the individual catalysts. In order to investigate the influence of a gas mixture that more closely reflects the real engine exhaust conditions, the experiments are carried out in the presence of carbon monoxide (CO), nitrogen monoxide (NO), and carbon dioxide (CO₂). To simulate the DME blend exhaust gases, a propene concentration of c_C3H6_ = 250 ppm is introduced, analogous to the approach employed for gas mixtures 3 and 4. Propene has previously proven effective as a surrogate for diesel exhaust in studies involving diesel oxidation catalysts (DOCs) [10]. A comprehensive list of all test parameters can be found in Table 2. Gas mixture 5 is exclusively employed for catalysts DOC 1, DOC 3, and DOC 5 to explicitly compare and validate the influence of DME on NO oxidation against the documented effect of propene on NO oxidation in existing literature.

**Table 2:** Experimental conditions of the conducted Light-Off experiments at the laboratory gas test bench.

	O_2_	DME	C_3_H_6_	CO	NO	CO_2_	H_2_O	N_2_
	vol.- %	ppm	ppm	ppm	ppm	vol.- %	vol.- %	-
**Gas Mixture 1: DME reduced**	10	500	0	0	0	0	8	Rest
**Gas Mixture 2: DME realistic**	10	500	0	700	500	6	8	Rest
**Gas Mixture 3: Diesel/DME blend reduced**	10	500	250	0	0	0	8	Rest
**Gas Mixture 4: Diesel/DME blend realistic**	10	500	250	700	500	6	8	Rest
**Gas Mixture 5: Zero HC reference**	10	0	0	700	500	6	8	Rest

The concentrations contained therein are derived from empirical values based on data from the Ford-RWTH Alliance project “Emission Control System for e-Fuels,” in which, among other things, DME was used as a pure fuel under different operating conditions.

At the beginning and end of each experiment, the dosage and measurement of each gas component are meticulously checked. To this end, the catalyst sample installed in the SGB is circulated through a bypass and measured directly in the analyzers. As depicted in Fig. 2, a standard exhaust gas line by FEV Europe GmbH, combining a non-dispersive infrared detector (NDIR), a paramagnetic detector (PMD), and a flame ionization detector (FID) are used to detect CO, CO_2_, O_2_ and the total hydrocarbons (THC). This line also implements a Multigas 2030 HS FTIR from MKS Instruments for measuring CO, CO_2_, NO_x_, H_2_O, DME and other hydrocarbon species. The online H_2_ measurement is done by an electron impulse mass spectrometer (EIMS), HSense by V&F Analyse und Messtechnik GmbH. The utilization of these individual measuring devices is predicated on the determination of the concentration of the most salient substances. All analytic devices are calibrated daily with their respective species from certified gas cylinders and the FTIR is flushed with N_2_ in 5.0 quality for a background check.

**Fig. 3:**
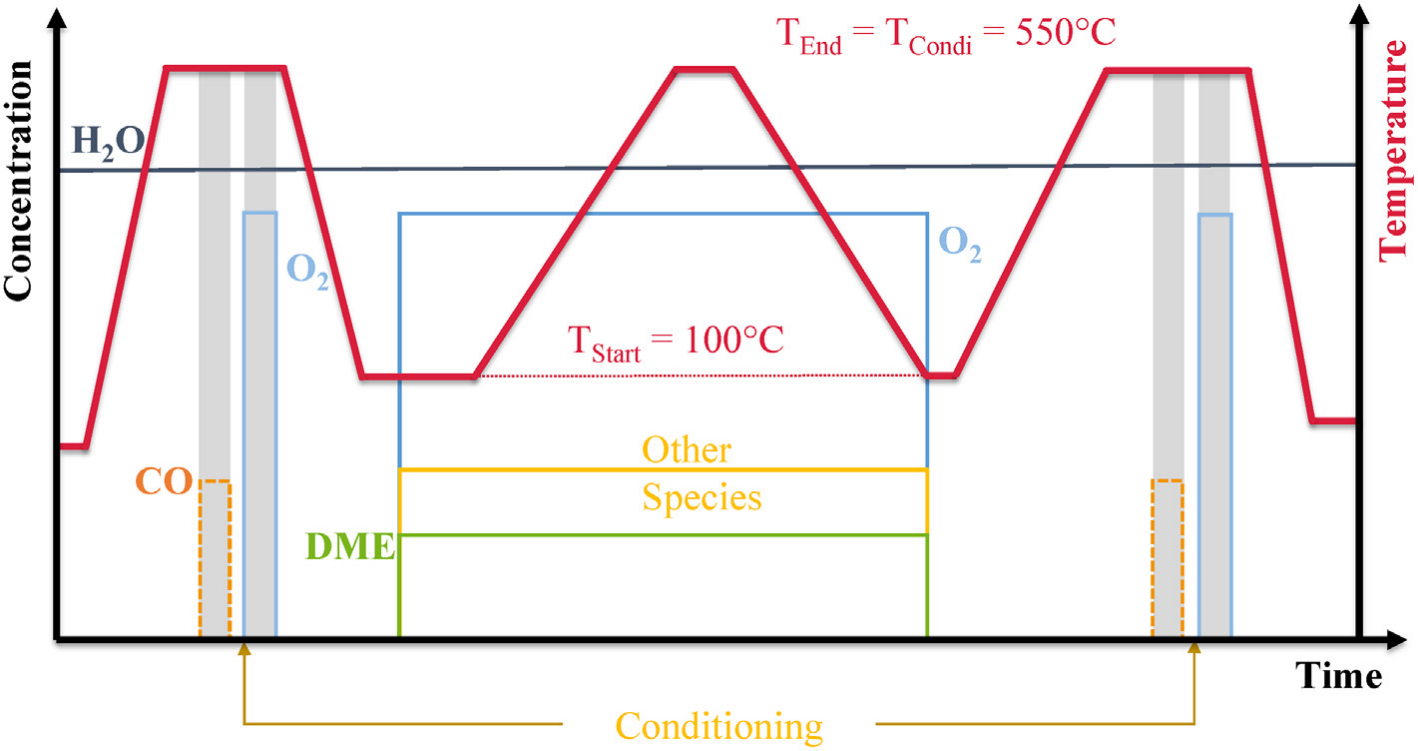
Schematic representation of the test procedure for analyzing the DME conversion characteristics.

Fig. 3 presents the schematic test sequence of a light-off measurement. To ensure the reproducibility of measurements, the catalyst sample is preconditioned at the initiation of the measurement process. To this end, the sample is heated to a temperature of T = 550 °C using an automated temperature ramp (gradient δ_T_/δ_t_ = 10 K/min) and a gas flow consisting of c_H2O_ = 8 vol.- % in N_2_. Once the desired temperature has been attained, the conditioning gas matrix - consisting of c_CO_ = 700 ppm and c_H2O_ = 8 vol.- % in N_2_ - is introduced to the catalyst for a duration of t = 5 min. Subsequent to this reduction, the catalyst sample is subjected to the second conditioning gas matrix, comprising oxygen with c_O2_ = 10 vol.- % and c_H2O_ = 8 vol.- % in N_2_, for a duration of t = 5 min. Subsequent to preconditioning, the sample is cooled to the designated test temperature of T = 100 °C, with a H_2_O content of c_H2O_ = 8 vol.- % in nitrogen.

Upon attaining the designated test temperature, the administration of gas mixtures 1–5 commences, accompanied by the introduction of all other gas components. Subsequent to a stabilization period of t = 15 min, the automated temperature ramp, with a gradient of δ_T_/δ_t_ = 5 K/min, is initiated to achieve a target temperature of T = 550 °C, employing the same gas composition. Subsequent to attaining the maximum temperature, the temperature is sustained for a duration of t = 5 min and then undergoes a cooling process to the initial temperature of T = 100 °C via an automated temperature ramp. Subsequent to attaining the initial temperature, the administration of all reaction gases is ceased, and the sample is heated once more to the conditioning temperature with a gas composition of c_H2O_ = 8 vol.- % in N_2_ and a ramp with a gradient of δ_T_/δ_t_ = 10 K/min. The application of heat in water and nitrogen allows conclusions in regard to unreacted and stored components. Subsequent to the completion of thermal desorption, the sample undergoes a conditioning procedure, akin to the initial phase of the measurement.

### 4.3 Post processing and data acquisition

The efficient management and analysis of experimental data are crucial for obtaining reliable and repeatable results. This section discusses functionalities of the test bench software, based on LabVIEW (release 2011), which plays a vital role in monitoring and controlling various parameters during the experiments.

The software outputs all relevant setpoints and return values from mass flow controllers, temperature readings of all measurement points, control parameters for temperature regulation, and recorded exhaust measurement data. Initially, these data must undergo evaluation to derive values for flow rates and concentrations. The mass flow controllers operate within a voltage range of 0–5 V for both input and output signals, which are stored in raw measurement files. From these voltage values, the dosed gas concentrations at the inlet of the sample can be calculated. To ensure accurate correlations between mass flow controllers, their control values, and the concentrations from the used gas cylinders, all relevant informations are stored in a self-developed SQL database for each experiment. Additionally, calibration curves in N_2_ and corresponding correction factors for the dosed test gases are also documented for each MFC. These data enable the conversion into the desired dosed concentrations during, both planning and evaluation phases. Data evaluation is conducted using a custom script, programmed in Visual Basic that accesses all necessary experimental data stored within the database. This evaluation script computes all concentrations and key characteristics pertinent to the areas of interest within the experiments. Furthermore, measured values undergo dead time correction and zero-point adjustment to account for gas transit times and potential drifts among various measuring devices.

In conclusion, this integrated approach to data processing ensures that laboratory measurements yield reliable results while facilitating comprehensive evaluations critical for advancing research objectives. For an accuracy measure of the T_50_ and X_50_ evaluations regarding measurement noise from gas analytics and gas dosing via MFCs, the noise in the calculated DME conversion is evaluated against the measurement range. This is exemplified by the four experiments of DOC 3. Since the dataset does not use data smoothing for gas analytics or MFC feedback values, the calculated DME conversion shown in Eq. (1) is subject to overlaying noise. For analysis, the noise interval is assessed for X_50_DME_ during both inclining and declining temperature ramps by setting the interval from the lowest temperature over X_50_DME_ to the highest temperature under X_50_DME_. The calculation accuracy of T_50_DME_ with the temperature range of T = 450 °C is ± 0.125 % and at the conversion range of 0 % to 100 % is 0.32 % for X_50_DME_. Combined with the MFCs accuracy of ± 0.5 %, this results in ± 0.63 % and ± 0.66 % respectively.

### 4.4 Catalyst specifications and sample preparation

The PGM loading characteristics of the investigated catalysts are summarized in Table 3. These state-of-the-art catalysts are bimetallic DOCs derived from series applications, with platinum content exceeding that of palladium across all samples. The loading for DOC 5 remains unknown; however, it is presumed to be a cold start catalyst optimized for cold start performance.

**Table 3:** PGM loading of the investigated catalysts; DOC 1–5.

Sample	Loading	Pt/Pd-Ratio
-	g/ft^3^	g_Pt_/g_Pd_
**DOC 1**	90	4 / 1
**DOC 2**	90	3 / 1
**DOC 3**	105	6.2 / 1
**DOC 4**	94	4 / 1
**DOC 5**	unknown	unknown

All catalyst samples are drilled from full monolithic substrates with a diameter of D_Sample_ = 19 mm. The volume of each sample allows for calculations based on the desired space velocity (GHSV = 120,000 h^-1^), enabling accurate flow rate determinations. All catalysts utilize monolithic substrates featuring a cell density of CPSI = 400 composed of cordierite material. Also, the preparation of the catalyst samples for testing includes a hydrothermal aging, always under the same conditions with c_H2O_ = 10 vol.- %, for T_Aging_ = 16 h.

**Fig. 4:**
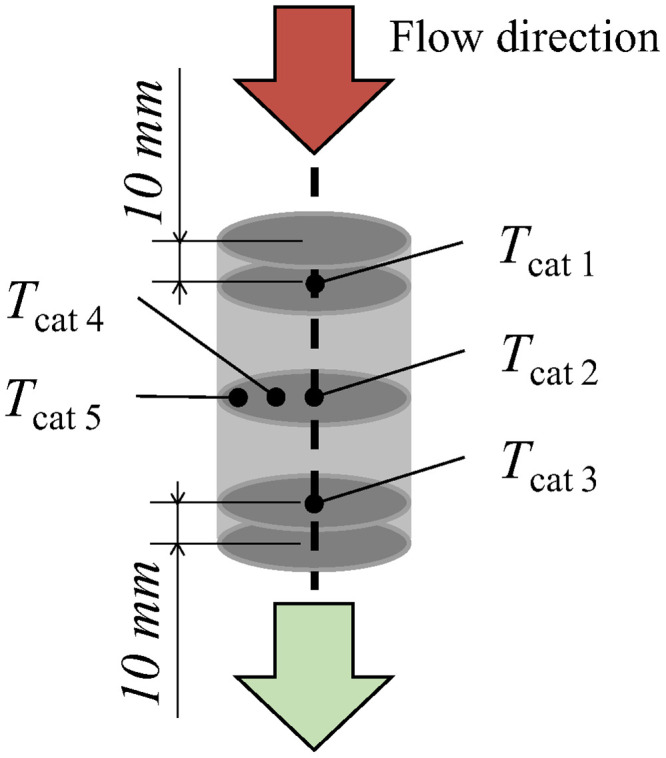
Positioning of the thermocouples within the catalyst sample for temperature measurement.

The catalyst samples are placed into the testbench via a titanium canning, where the catalyst is held in place by a swelling mat, wrapped around the sample. As illustrated in Fig. 4, the sample within the SGB is equipped with five type K thermocouples (TC) from Thermo Sensor GmbH. They have a diameter of Ø_TC_ = 0.5 mm and a length of l_TC_ = 750 mm, with a temperature range of T_TC_ = −40 °C … 1000 °C. The first three TC are positioned along the centerline of the sample, 10 mm subsequent to the inlet, at the center and 10 mm prior to the outlet. TC 4 and 5 are positioned at the midpoint of the sample, on a small and large radius respectively. This facilitates a precise evaluation of the respective exothermic or endothermic reactions potentionally occurring on different catalyst regions.

## Limitations

The measurement data of the gas analytics underlies the precision of the respective gas analyzer, mass flow controller and certificate of the gas cylinder. With regard to the FTIR data, a multitude of species are monitored and displayed subsequent to the internal interpretation of the generated spectra. During the measurement campaign, an in-house adapted calculation method is employed. This method was developed to evaluate real exhaust gas from oxygenated fuels. However, given the experiments at the SGB contain such a broad variation of species, there is a possibility of cross-influences or misinterpretations. To counteract this issue, all captured spectra are stored within a database and can be accessed whenever needed. This enables the subsequent recalculation of the FTIR data using alternative methods, in the event that the initial interpretation appears to be compromised. The captured spectra are not subject to publication, but they can be shared upon request.

## Ethics Statement

The authors that they have read and follow the ethical requirements for publication in Data in Brief and confirm that the current work does not involve human subjects, animal experiments, or any data collected from social media platforms.

## Credit Author Statement

**Tom Padeken:** Investigation, Conceptualization, Writing – original draft. **Alexander Lampkowski:** Investigation, Writing – review & editing. **Peter Mauermann:** Methodology, Conceptualization. **Werner Willems:** Writing – review & editing, Project administration. **Christian Nederlof:** Conceptualization, Writing – review & editing. **Björn Franzke:** Writing – review & editing. **Stefan Sterlepper:** Supervision, Writing – review & editing. **Bastian Lerheuer:** Supervision, Writing – review & editing. **Stefan Pischinger:** Writing – review & editing, Supervision.

## Data Availability

ZenodoDataset for a Measurement Campaign of DME Oxidation on Commercial Pt/Pd-Series Diesel Oxidation Catalysts (Original data).
